# Microglial Nox2 Plays a Key Role in the Pathogenesis of Experimental Autoimmune Encephalomyelitis

**DOI:** 10.3389/fimmu.2021.638381

**Published:** 2021-04-02

**Authors:** Chih-Fen Hu, San-Pin Wu, Gu-Jiun Lin, Chi-Chang Shieh, Chih-Sin Hsu, Jing-Wun Chen, Shih-Heng Chen, Jau-Shyong Hong, Shyi-Jou Chen

**Affiliations:** ^1^ Graduate Institute of Medical Sciences, National Defense Medical Center, Taipei, Taiwan; ^2^ Department of Pediatrics, Tri-Service General Hospital, National Defense Medical Center, Taipei, Taiwan; ^3^ Reproductive and Developmental Biology Laboratory, National Institute of Environmental Health Sciences, National Institutes of Health, Research Triangle Park, NC, United States; ^4^ Department of Biology and Anatomy, National Defense Medical Center, Taipei, Taiwan; ^5^ Institute of Clinical Medicine, National Cheng Kung University College of Medicine, Tainan, Taiwan; ^6^ Genomics Center for Clinical and Biotechnological Applications of Cancer Progression Research Center, National Yang Ming Chiao Tung University, Taipei, Taiwan; ^7^ Graduate Institute of Life Sciences, National Defense Medical Center, Taipei, Taiwan; ^8^ Neurobiology Laboratory, National Institute of Environmental Health Sciences, National Institutes of Health, Research Triangle Park, NC, United States; ^9^ Department of Microbiology and Immunology, National Defense Medical Center, Taipei, Taiwan

**Keywords:** NADPH oxidase, reactive oxygen species, experimental autoimmune encephalomyelitis, chemotaxis, microglia

## Abstract

While oxidative stress has been linked to multiple sclerosis (MS), the role of superoxide-producing phagocyte NADPH oxidase (Nox2) in central nervous system (CNS) pathogenesis remains unclear. This study investigates the impact of Nox2 gene ablation on pro- and anti-inflammatory cytokine and chemokine production in a mouse experimental autoimmune encephalomyelitis (EAE) model. Nox2 deficiency attenuates EAE-induced neural damage and reduces disease severity, pathogenic immune cells infiltration, demyelination, and oxidative stress in the CNS. The number of autoreactive T cells, myeloid cells, and activated microglia, as well as the production of cytokines and chemokines, including GM-CSF, IFNγ, TNFα, IL-6, IL-10, IL-17A, CCL2, CCL5, and CXCL10, were much lower in the Nox2^−/−^ CNS tissues but remained unaltered in the peripheral lymphoid organs. RNA-seq profiling of microglial transcriptome identified a panel of Nox2 dependent proinflammatory genes: *Pf4*, *Tnfrsf9*, *Tnfsf12*, *Tnfsf13*, *Ccl7*, *Cxcl3*, and *Cxcl9*. Furthermore, gene ontology and pathway enrichment analyses revealed that microglial Nox2 plays a regulatory role in multiple pathways known to be important for MS/EAE pathogenesis, including STAT3, glutathione, leukotriene biosynthesis, IL-8, HMGB1, NRF2, systemic lupus erythematosus in B cells, and T cell exhaustion signaling. Taken together, our results provide new insights into the critical functions performed by microglial Nox2 during the EAE pathogenesis, suggesting that Nox2 inhibition may represent an important therapeutic target for MS.

## Introduction

Multiple sclerosis (MS) is one of the major neurodegenerative diseases. Many drugs are available for MS therapy, but these treatments often do not effectively halt disease progression. Thus, the identification of disease-modifying drugs that can stop MS progression are urgently needed (1). Mouse experimental autoimmune encephalomyelitis (EAE) is widely used to model human MS (2). The key features of the mouse EAE model that somewhat recapitulate the several immunopathological and neuropathological features of human MS include inflammation, demyelination, axonal loss, and gliosis (3). While the roles performed by immune cells related to autoimmune functions [e.g. autoreactive CD4^+^ T cells (T_H_1 and T_H_17 cells), CD8^+^ T cells, memory B cells, and myeloid cells (monocytes, dendritic cells)] are well known (4), the influence of microglia on MS/EAE remains unclear.

Microglia are a major source of pro-inflammatory cytokines (e.g. TNFα, IL-1β, IL-6, IL-17, and IL-23) and chemokines (e.g. CCL2, CCL3, CCL4, CCL5, CCL12, and CCL22) that may worsen MS/EAE (5). Therefore, deregulated microglia can aggravate devastating demyelination and neuronal damage in the brains of MS patients (6). Microglial nodules, characterized by an absence of leukocyte infiltration, astrogliosis, or demyelination, in normal-appearing white matter of MS patient brains are linked to the earliest stage of MS lesion formation. Known as “pre-active lesions,” these clusters of activated microglia are believed to eventually develop into pronounced and active demyelinating MS lesions (7). Kinetics studies from EAE mouse models suggest that microglia are immediately activated by MOG treatment and represent the earliest cell population to take up myelin antigens (8). Subsequently, through major histocompatibility complex (MHC) molecules, these microglia re-stimulate and recruit autoreactive T cells into the central nervous system (CNS) (9).

Microglia-generated superoxide and its immune cell-produced metabolites play a critical role in the progression of various neurodegenerative disorders, including MS/EAE (10, 11). Nicotinamideadenine-dinucleotide phosphate (NADPH) oxidase (Nox2) is a key superoxide-producing enzyme that forms reactive oxygen species (ROS) (12). Nox2 is highly expressed in professional phagocytes (e.g. neutrophils, monocytes, macrophages, microglia, and dendritic cells). Nox2 is critical for host defense because it produces reactive oxidants, activates granular proteases, and assists in the generation of neutrophil extracellular traps. Nox2 is the catalytic, membrane-bound subunit of NADPH oxidase. It is composed of an N-terminal transmembrane domain that interacts with two heme groups and a C-terminal domain that associates with both flavin adenine dinucleotide and NADPH (13). Nox2 remains inactive until it interacts with the membrane-anchored p22*phox*, resulting in the formation of the flavocytochrome b558 heterodimer (14). After activation by various infectious agents, the regulatory subunits p67*phox*, p47*phox*, p40*phox*, and the small Rho GTPase Rac are recruited to the complex to form NADPH oxidase on the plasma or phagosomal membrane (15). Here, Nox2 converts molecular oxygen into superoxide anions, which can be further transformed into antimicrobial metabolites (e.g. hydroxyl anion and hydrogen peroxide) (16). Thus, Nox2 is a major contributor to oxidative stress and subsequent neurotoxicity in the CNS (17). However, the mechanisms underlying Nox2-dependent MS pathogenesis remain unknown. Here, we sought to address this gap in our understanding of MS/EAE pathogenesis by investigating the contribution of Nox2 activity to myelin oligodendrocyte glycoprotein (MOG)-elicited EAE.

In this study, we provide strong evidence indicating that microglia play a critical role in the induction of EAE pathologies. Nox2-generated superoxide and excessive oxidative stress as well as Nox2-mediated chemotaxis in microglia are both essential mediators to cause advanced demyelination of oligodendrocytes and damage of neurons. Furthermore, our data also suggests Nox2 is prime target for developing therapy for MS/EAE.

## Materials and Methods

### Animals

C57BL/6 gp91^phox−/−^ [Nox2 KO mice (gp91^Cybbtm1Din/J^)] mice (18) were kindly provided by Dr. Chi-Chang Shieh (Institute of Clinical Medicine, National Cheng Kung University College of Medicine, Tainan city, Taiwan). Control mice, C57BL/6 gp91^phox+/+^ (wildtype mice), were purchased from National Laboratory Animal Center in Taipei City, Taiwan. Both mouse strains were subsequently bred at the Animal Center of the National Defense Medical Center under pathogen-free conditions. Mice were treated in accordance with the Institutional Animal Care and Use Committee of the National Defense Medical Center guide for experiments and approved by the committee in the same office (IACUC: 19-058).

### Chronic EAE Induction and Disease Score

Mice were given a subcutaneous injection of 100 μg of MOG_35–55_ emulsified with complete Freund’s adjuvant (MOG/CFA) which contained 0.8 mg of heat-inactivated *Mycobacterium tuberculosis* (H37RA; Difco Laboratories) in their flanks. Each animal also received pertussis toxin (19) (500 ng i.p.; List Biological Laboratories) on day 0 and day 2 post-immunization. Mice were monitored daily up to 42 days for signs of disease. The EAE clinical score was determined based on the following scale: 0: normal mouse, no overt signs of disease; 1: limp tail or hind limb weakness, but not both; 2: limp tail and hind limbs weakness or poor balance or head tilting when walking; 3: Limp tail and complete paralysis of hind limbs or one front and one hind limb; 4: Limp tail, complete hind limbs and partial front limbs paralysis; 5: moribund state or death. For our pathologic score, the scale ranges from 0 to 5 and represents normal to most severe immune cells’ infiltration at the myelin area. 0: no infiltration, 1: ≤10%, 2: 11–20%, 3: 21–30%, 4: 31–40%, 5: >40% (reviewed by a neuropathologist). In-between scores (i.e. 0.5, 1.5, 2.5, 3.5, 4.5) when the clinical or pathologic picture lies between two defined scores.

### Isolation of Splenocytes, Lymph Node Cells, and CNS Mononuclear Cells

Cells were isolated from mouse spleen and cervical lymph node by mashing tissues between two frosted microscope slides. The cells were further treated with RBC lysis buffer (Gibco, catalog number: A1049201) to eliminate erythrocytes, washed, and resuspended in RPMI 1640 (Gibco, catalog number: 31800022) supplemented with 10% FBS, 2 mM L-glutamine, 100 U/ml penicillin G, 0.1 mg/ml streptomycin, and 10 mM HEPES (Life Technologies, Waltham, MA, USA). Isolation of CNS mononuclear cells was achieved using Percoll-gradient separation (GE Healthcare Bio-Sciences, Uppsala, Sweden) as previous described (20).

### Isolation of CD11b^+^CD45^int^Tmem119^+^ Microglia From CNS Mononuclear Cells

Live CD11b^+^CD45^int^Tmem119^+^ microglia were isolated by cell sorting using a FACSAria Fusion (BD Biosciences, USA). After sorting, we sampled 300 cells (by the flow cytometry) for purity check to make sure the population is >95% microglia.

### Hematoxylin and Eosin Stains and Immunofluorescence

Mouse lumbar spinal cord sections were used for hematoxylin and eosin staining (H&E Staining Kit; Abcam, catalog number: ab245880), single myelin staining (FluoroMyelin Green Fluorescent, 1:300; Invitrogen, catalog number: F34651), and triple-labeled immunofluorescence. Before primary antibody conjugation, additional blocking with mouse-on-mouse blocking reagents (Vector lab, catalog number: R37621) was performed on each sample. 3-NT antibody (1:1,500; Abcam, catalog number: ab61392), in combination with antibody specific for CD11b (1:1,500; Bio-Rad, catalog number: MCA711G), ASPA (1:200; Millipore, catalog number: ABN1698), Neu-N (1:1,500; Abcam, catalog number: 177487), Iba-1 (1:1,500; WAKO, catalog number: 019-19741), or GFAP (1:1,500; DAKO, catalog number: 00087880), were used as indicated in the figure legends. Nox2 antibody (1:1,500; BD biosciences, catalog number: 611415) was counterstained with CD45 (1:1,500; BioLegend, catalog number: 103101), CD16/32 (1:1,500; BioLegend, catalog number: 101301), Iba-1 or Neu-N. Phosphorylated p65 antibody (1:500; Millipore, catalog number: 3015337) was used to detect NF-κB activation in combination with CD11b and Neu-N. Spinal cord sections were then incubated with Alexa-488 (1:1,500; Invitrogen, catalog number: A32731), Alexa-568 (1:1,500; Invitrogen, catalog number: A-11011), and Alexa-647 (1:1,500; Invitrogen, catalog number: A32733) conjugated secondary antibodies and mounted with DAPI (Vector lab, catalog number: 62248). The digital scanning analysis were kindly provided by the CMPB Image Analysis Lab at the NIEHS (LEICA-Scan scope FL System). All fluorescent images were obtained with a Zeiss LSM 780 laser scanning confocal microscope. Data were analyzed by Zen black version 2.0.

### *In Vivo* Imaging System (IVIS)

*In vivo* imaging was performed in mice after EAE induction to measure the levels of active myeloperoxidase (MPO) in activated phagocytes non-invasively. Before study, all the mice received hair removal at areas of interest to reduce the interreference of the desired signal. Anesthesia was induced with 2% isoflurane (Abbott Laboratories) inhalation in a special air tight transparent anesthesia box for 3–5 min before the mice were moved to the light-tight chamber of the CCD camera in the imaging position. Bioluminescent images of inflammation at CNS area and MOG inoculation site were taken 10 min post intraperitoneal injection of the inflammation probe (XenoLight RediJect, PerkinElmer, 200 mg/kg) with IVIS Spectrum (PerkinElmer, 5 min of exposure time). XenoLight RediJect Inflammation Probe is a ready-to-use chemiluminescent reagent and can be conveniently applied to study MPO activity of activated phagocytes. RediJect D-Luciferin (K^+^ salt) is a bioluminescent *in vivo* substrate in a ready-to-use pre-formulated injectable format as a Luciferin-based conjugates as the bioluminescent imaging probe. The luminescence camera was set to 60 s exposure, medium binning, f/1, blocked excitation filter, and open emission filter. The photographic camera was set to 2 s exposure, medium binning, and f/8. Field of view was set to image all mice simultaneously. Identical settings were used to acquire each image and region of interest during the study as previously described. The luminescent areas of the CNS region and MOG inoculation site were defined as the region of interest (ROI) and the total signal in the ROI (photon/sec/m^2^) was quantified using Living Image software 3D (version: 4.4.17197; PerkinElmer).

### Flow Cytometry for T Cells and Myeloid Cells Analysis From Splenocytes, Cervical Lymphoid Cells, and CNS Mononuclear Cells

Before cytokine staining, T cells underwent *in vitro* stimulation with PMA (40 ng/ml), Ionomycin (2 μM), Monensin (4 μM) in 1 ml RPMI 1640 (containing 10% FBS, 2 mM L-glutamine, 100 U/ml penicillin G, 0.1 mg/ml streptomycin, and 10 mM HEPES) for 4 h. Cells were resuspended in PBS supplemented with 0.5% BSA and 1 mM EDTA (FACS buffer) (splenocytes and lymphoid cells: 1 × 10^6^/tube, all available CNS mononuclear cells from each sample/tube) and stained with the antibodies listed below at 4˚C for 30 min. The anti-mouse antibodies used for flow cytometric analysis were the following: anti–CD45–APC-Cy7, anti-CD4-APC, anti-IFNγ-BV421, anti-IL-17–PerCP Cy5.5, anti-IL-10–FITC, anti-Foxp3-PE, anti-CD11b-PE-Cy7, anti-Ly6G-BV421, anti-MHC II-FITC, anti-Ly6C-PerCP Cy5.5, anti-CCR2- BV510 (all antibodies were from eBiosciences or BioLegend). After staining, the cells were analyzed with FACSVerse flow cytometer. Flow cytometric data were viewed and analyzed by FlowJo v10.

### RNA Extraction, Reverse Transcription, and Quantitative Real-Time PCR

Homogenized mouse spinal cords (L4-spine level) or primary microglia culture were lysed with 1 ml TRIzol Reagent (Invitrogen). Nucleoprotein complexes were then separated through bromochloropropane (Sigma-Aldrich), and the samples were centrifuged at 12,000 rpm at 4˚C for 15 min. Total RNA was precipitated with an equal volume of isopropanol. The samples were incubated at 25˚C for 10 min and centrifuged at 12,000 rpm at 4˚C for 15 min. The RNA pellets were collected and washed with ethanol by vigorous mixing. RNA was obtained by centrifugation at 7,500 rpm at 4˚C for 10 min. After air-drying, the RNA pellets were dissolved in RNase-free double-distilled water. Reverse transcription was performed using a Reverse Transcription Kit (Applied Biosystems) to synthesize cDNA. Quantitative real-time PCR (qPCR) was performed using FastStart Universal SYBR Green Master with ROX (Roche), and cDNA was amplified using a StepOnePlus RealTime PCR System (Applied Biosystems). PCR cycling conditions were set as follows: 95°C for 10 min, then 40 cycles of 95°C for 15 s, 60°C for 1 min, and 72°C for 20 s. The specificity of the SYBR green assay was confirmed by melting-point analysis. Expression data was calculated from the cycle threshold (Ct) value using the ΔCt method for quantification. Primer sequences are listed in **Supplementary Table 1**.

### Primary Cell Cultures

Primary splenocytes culture for ELISA: 1 × 10^6^/well, in 48-well plate, with or without MOG 10 μg/ml in 500 μl RPMI 1640 (10% FBS, 2 mM L-glutamine, 100 U/ml penicillin G, 0.1 mg/ml streptomycin, and 10 mM HEPES) for 72 h. Primary CNS mononuclear cells culture for ELISA: 1.5 × 10^5^/well, in 48-well plate, with or without MOG 10 ug/ml in 500 μl DMEM/F12 (10% FBS, 2 mM L-glutamine, 100 U/ml penicillin G, 0.1 mg/ml streptomycin, and 10 mM HEPES) for 72 h. Primary microglia culture (CD11b^+^CD45^int^Tmem119^+^) for RNA-sequencing and ELISA: 3 × 10^4^/well, in 48-well plate, overnight seeding (18 h), then added MOG 10 μg/ml in 500 μl DMEM/F12 (10% FBS, 2 mM L-glutamine, 100 U/ml penicillin G, 0.1 mg/ml streptomycin, and 10 mM HEPES) for 24 h.

### Enzyme-Linked Immune Absorbent Spot (ELISpot)

The frequency of IFNγ, IL-4, and IL-17–producing cells on preclinical (D7) and disease peak (D17) stage at the spleen were determined using ELISpot kit (Immunospot, C.T.L.) according to the manufacturer’s instructions. Splenocytes (5 × 10^5^/well, in 200 ul) were plated in an ELISpot plate coated with rat anti-mouse IFNγ, IL-4, and IL-17 capture antibodies in the presence of 10 μg/ml MOG or PMA (2 mg/ml) plus Ionomycin (0.1 μg/ml) or no *in vitro* stimulation. Cells were cultured overnight and IFNγ, IL-4, and IL-17 production were detected using a biotin-conjugated rat anti-mouse IFNγ, IL-4, and IL-17 detection antibodies. Avidin-HRP was then added, and spots were developed with 3-amino-9-ethylcarbamazole. Specific IFNγ, IL-4, and IL-17 production were calculated by the CTL ImmunoSpot^®^ Analyzers along with the ImmunoSpot^®^ software provided automated, objective recognition of spots, gating, and counting.

### Multiplex Enzyme-Linked Immunosorbent Assay (ELISA)

Plasma samples were analyzed by multiplex enzyme-linked immunosorbent assay (mouse cytokine/chemokine magnetic bead panel, MCYTOMAG-70K, EMD millipore). An array of 11 cytokines/chemokines was analyzed: GM-CSF, IFNγ, TNFα, IL-1β, IL-6, IL-10, IL-12p70, IL-17A, CCL2, CCL5, and CXCL10 using a Luminex—MAGPIX^®^ System and MILLIPLEX Analyst 5.1 software. Assay Sensitivity: GM-CSF: 2.36 pg/ml; IFNγ: 1.04 pg/ml; TNFα: 3.04 pg/ml; IL-1β: 2.97 pg/ml; IL-6: 2.89 pg/ml; IL-10: 2.68 pg/ml; IL-12p70: 3.40 pg/ml; IL-17A: 1.21 pg/ml; CCL2: 1.13 pg/ml; CCL5: 2.64 pg/ml; CXCL10: 2.20 pg/ml.

### RNA-Seq Library Preparation and Sequencing

Total RNA was subjected to cDNA synthesis and NGS (NextGen Sequencing) library construction using the Ovation SoLo RNA-Seq System (NuGEN Technologies, Redwood City, CA, USA). The quality and average length of sequence library for each sample was assessed using Bioanalyzer (Agilent Technologies, Santa Clara, CA, USA) and either the DNA 1000 kit. The indexed samples were pooled equimolarily and sequenced on Illumina NovaSeq 6000 (150 bp, paired-end reads) (Illumina, San Diego, CA, USA).

### Bioinformatics Analysis

The quantification of raw RNAseq reads was processed using CLC Genomics Workbench v.10 software. Adaptor sequences and base with low quality or ambiguous were trimmed. The quality screened reads were mapped to mouse (GRCm38) genome using CLC Genomics Workbench. The mapping parameters were the following: mismatch cost 2, insertion cost 3, deletion cost 3, length fraction of 0.5, and similarity fraction of 0.8. The expression values were calculated as FPKM (Fragments Per Kilobases per Million). The differential gene expression between two or more condition based on the fold change of FPKM value. The genes with 2-fold change were further analyzed. The total transcripts from three samples (K-pooled, W1, and W2), 46,202, were filtered with protein-coding region first and left the items with either FPKM > 1 among these three samples. KEGG database (21, 22) was used in pathway enrichment analysis and the pathway map was plotted by pathview (23) package in R. Gene set enrichment analysis and Gene ontology enrichment analysis were done with the GSEA/MSigDB (24) and GO-TermFinder (25), respectively. Ingenuity Pathway Analysis (IPA, Qiagen, Germantown, MD, USA) was used to search for enriched canonical pathways.

### Statistical Analysis

Prism v5.03 software (GraphPad Software, San Diego, CA, USA) was used to generate graphs and for statistical analysis. Student unpaired t test was used for statistical analysis of the experiments in this study. All figures are presented as mean ± SEM. A *p* value 0.05 was defined as significant (*p < 0.05, **p < 0.01, ***p < 0.001; ns, not significant).

## Results

### Nox2 Deficiency Ameliorates EAE-Elicited Disease Severity, Body Weight Loss, Leukocyte Infiltration, and Demyelination

**Figure 1:**
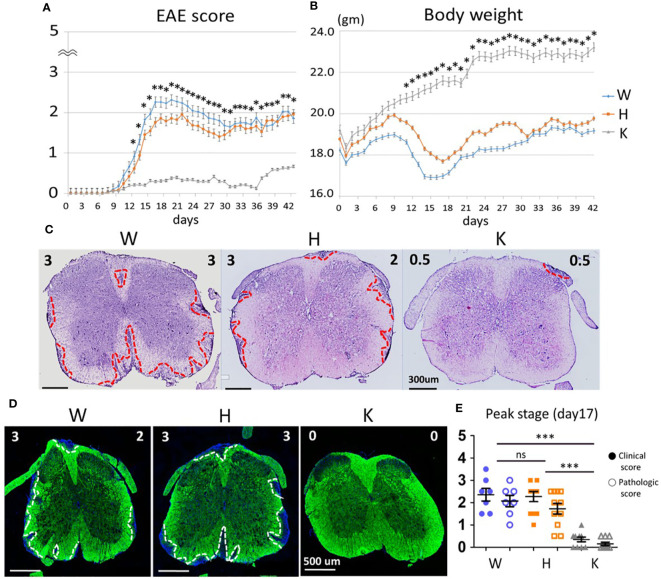
Nox2-deficient mice show less inflammatory infiltration, demyelination at lumbar spinal cord after EAE induction on disease peak (17 dpi). **(A)** EAE disease score and **(B)** Body weight change among three genotypes (mean ± SEM) [n = 33 (W), 63 (H), 46 (K)]. **(C)** Representative Hematoxylin and Eosin stains and **(D)** Fluoromyelin staining of lumbar spinal cord at the peak of EAE score (17 dpi). Left upper corner score: clinical score. Right upper corner score: pathologic score. The red dot lines outline the areas of leukocytes infiltration while the white dot lines outline the areas of demyelination. **(E)** Clinical score and pathologic score on day 17 based on the results of H&E stains [n = 7 (W), 11 (H), 10 (K)]. Genotype: W, *Nox2* ^+/+^; H, *Nox2* ^+/−^; K, *Nox2* ^−/−^. Formalin-fixed frozen section: 10 μm; spinal cord level: lumbar fourth, myelin: green; DAPI: blue. *p* value: *** < 0.001, * < 0.05; ns, not significant.

To investigate the role of Nox2 activity during EAE pathogenesis, we utilized three genotypes of female mice including *Nox2* ^+/+^ (W), *Nox2* ^+/−^ (H), *Nox2* ^−/−^ (K) to determine the disease score and body weight changes (**Figures 1A, B**). The W and H mice showed similar time-related changes in clinical phenotype and symptoms onset beginning on 9~10 days post-injection (dpi) and then reached peak stage at 17 dpi, while the K mice remained almost symptomless throughout the experiment. The severity of the EAE was evaluated by the degree of leukocyte infiltration (hematoxylin and eosin staining) and demyelination (fluoromyelin staining) in spinal cord sections (L4 level) from each mouse strain. The dashed red lines outline the leukocyte invasion area and the dashed white lines mark the contour of demyelination location. These results showed that the W and H mice exhibited similar patterns of leukocyte infiltration and demyelination, whereas no clear lesions were observed in K mice (**Figures 1C, D**). In addition, the staining sections were evaluated blindly by a neuropathologist to give a quantitative score of infiltration and (0: normal, 5: most severe). Comparison of the EAE score and the pathologic score on disease peak (17 dpi) showed that these two scoring systems were well correlated (**Figure 1E**). In conclusion, Nox2-deficient mice show less inflammatory infiltration, demyelination at lumbar spinal cord after EAE induction on disease peak (17 dpi) compared with wildtype mice.

### Nox2 Deficiency Greatly Reduces EAE-Elicited MPO Activity Within the CNS and MOG Inoculation Areas

**Figure 2:**
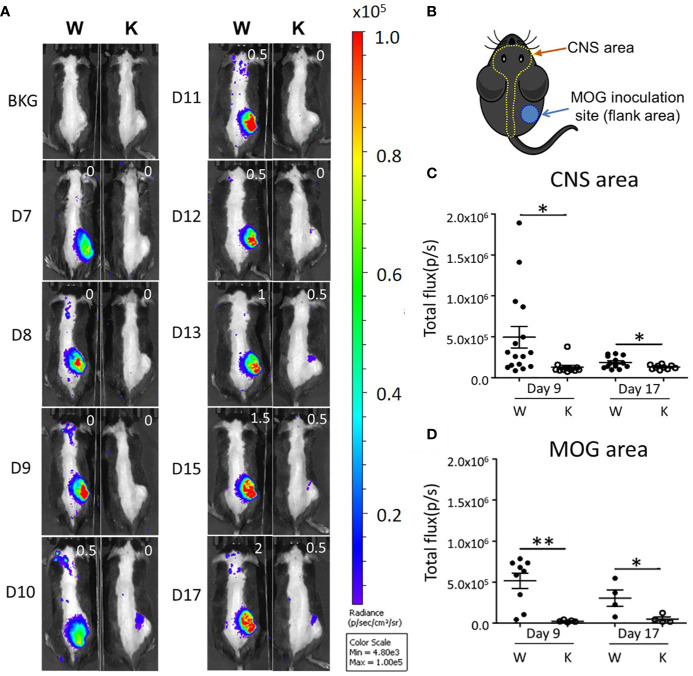
*In vivo* imaging analysis shows that EAE induction enhances MPO activity in both the CNS and MOG inoculation areas. **(A)** Serial daily imaging analysis showing the MPO signal at CNS and MOG inoculation areas. Right upper corner shows clinical EAE score. **(B)** Graph showing the shaved CNS and MOG inoculation areas for the detection of chemiluminescent signal by the IVIS Spectrum. **(C)** Early and **(D)** peak stages imaging: the MPO signal (mean ± SEM) from CNS and MOG inoculation areas on early disease stage (9 dpi) and peak stage (17 dpi) respectively. CNS area: day 9: n = 16 (W), 12 (K); day 17: 13 (W), 10 (K). MOG area: day 9: n = 9 (W), 5 (K); day 17: 4 (W), 4 (K). Genotype: W, *Nox2* ^+/+^; K, *Nox2* ^−/−^. *p* value: ** < 0.01, * < 0.05.

For longitudinal tracking of phagocyte-mediated inflammatory status (26), we injected the different mouse strains with the chemiluminescent XenoLight RediJect Inflammation Probe to measure MPO activity in activated phagocytes after EAE induction (**Figure 2A**). Bioluminescence here was used to assess the dynamic change of inflammation from CNS and MOG injection areas *in vivo*. Before being injected, mice were shaved at CNS and MOG inoculation areas for better signal detection (**Figure 2B**). Daily imaging analysis showed that the MPO signal present at the CNS area started to emerge at 8 dpi and was sustained up to 17 dpi in control animals, while almost no signal was detected in Nox2-deficent mice at these times (**Figures 2A**). Quantitative data on early (9 dpi) and peak disease (17 dpi) stage were shown on **Figures 2C, D**. In summary, Nox2-deficient mice show greatly reduced MPO signal at both CNS and MOG injection areas compared with wildtype mice which implies less oxidative stress and neuroinflammation status from early through peak disease stages.

### Nox2 Deficiency Specifically Reduces the EAE-Elicited Invasion of Immune Cells Into the CNS

**Figure 3:**
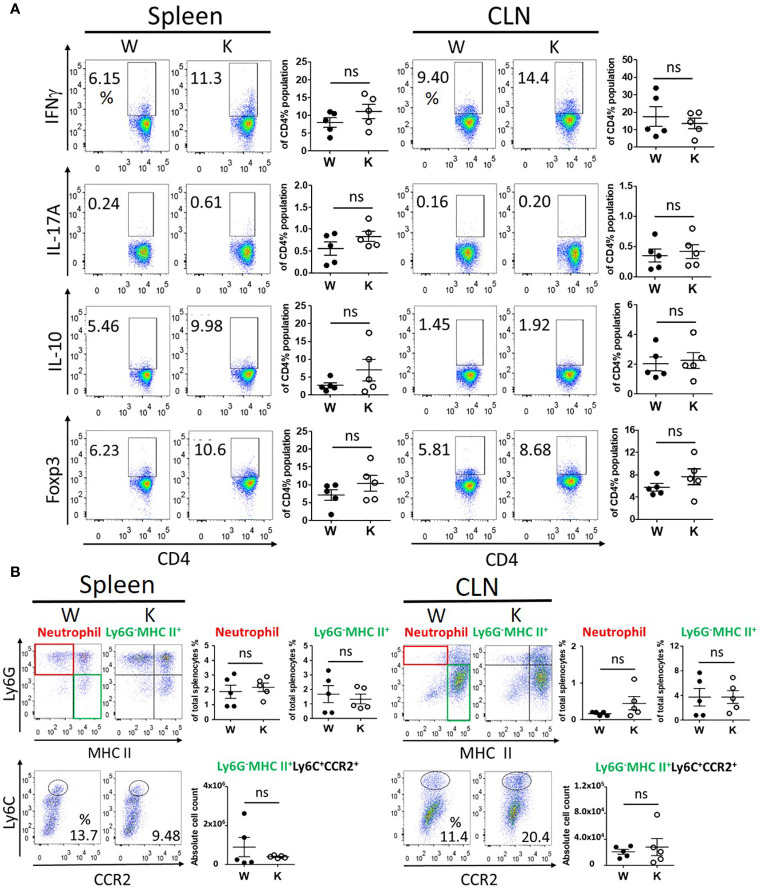
Nox2-deficient group presented similar profile of peripheral immune response as Nox2-competent group in spleen and cervical lymph nodes on disease peak (17 dpi). **(A)** the T cell profiles of IFNγ (T_H_1), IL-17A (T_H_17), IL-10, and Foxp3 (Treg)-producing cells from splenocytes (spleen) and cervical lymph nodes (CLN) respectively between W and K groups (n = 5 each group). **(B)** the myeloid cell profiles (CD11b^+^CD45^hi^) of neutrophils (Ly6G^+^MHC II^−^), monocytes (Ly6G^-^MHC II^+^), and cell count of pathogenic monocytes (Ly6G^-^MHCII^+^Ly6C^+^CCR2^+^) from spleen and CLN respectively between W and K groups (n = 5 each group). *p* value: ns, not significant.

**Figure 4:**
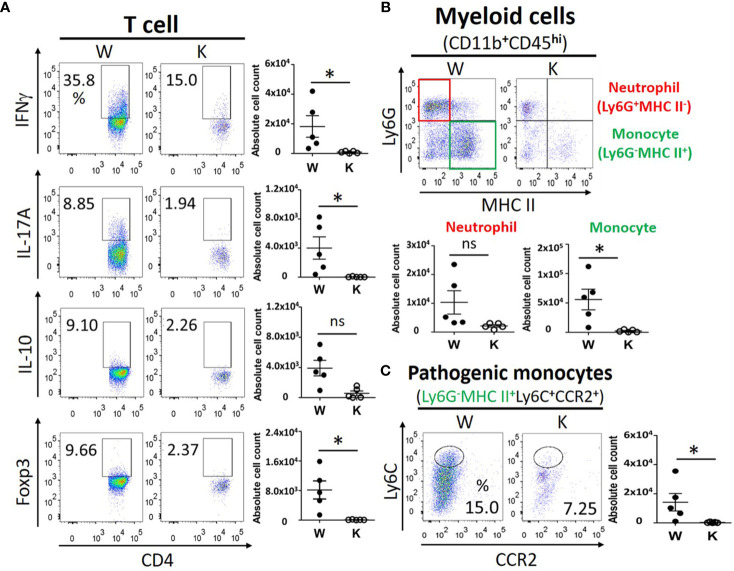
Nox2 deficiency greatly reduces the EAE-elicited invasion of pathogenic immune cells into the CNS on disease peak (17 dpi). **(A)** the T cell profiles of IFNγ (T_H_1), IL-17A (T_H_17), IL-10, and Foxp3 (Treg)-producing cells from CNS mononuclear cells between W and K groups (n = 5 each group). **(B)** the myeloid cell profiles (CD11b^+^CD45^hi^) of neutrophils (Ly6G^+^ MHC II^−^), monocytes (Ly6G^−^MHC II^+^), and **(C)** the absolute cell counts of pathogenic monocytes (Ly6G^−^MHCII^+^ Ly6C^+^CCR2^+^) from CNS mononuclear cells between W and K groups (n = 5 each group). *p* value: * < 0.05; ns, not significant.

To determine the immune response and immune cells population among peripheral lymphoid organs and CNS, we evaluated the proliferation and activation of T cells, neutrophils, and monocytes in the spleen, the cervical draining lymph nodes, and the CNS of each mouse strain at 17 dpi to study the distribution of pathogenic immune cells. No significant difference was observed between these mice strains regarding the levels of IFNγ (T_H_1)-producing T cells, IL-17A (T_H_17)-producing T cells, or IL-10- and Foxp3-producing regulatory CD4^+^ T cells in their spleens or cervical draining lymph nodes (**Figure 3A**). In contrast, the number of these T cells present within the CNS was reduced in the Nox2-deficinet mice relative to controls (**Figure 4A**). We also were unable to detect differences in the number of neutrophils (Ly6G^+^ MHC II^−^), monocytes (Ly6G^−^MHC II^+^), or pathogenic monocytes (Ly6G^−^MHCII^+^ Ly6C^+^CCR2^+^) (27) that infiltrated the spleen or cervical lymph nodes of the W and K mice (**Figure 3B**). However, the number of all these cell populations was greatly decreased in the CNS in Nox2-deficient mice (**Figures 4B, C**). Consequently, the peripheral T cells and myeloid cells profile are similar at both groups, but great reduction was observed in Nox2-deficient mice in CNS on disease peak, which implies that peripheral immune cells are already boosted and activated, but retained in the periphery rather than entering CNS.

### Nox2 Deficiency Specifically Decreases the EAE-Elicited Increase in the Levels of Cytokines and Chemokines Present Within the CNS

**Figure 5:**
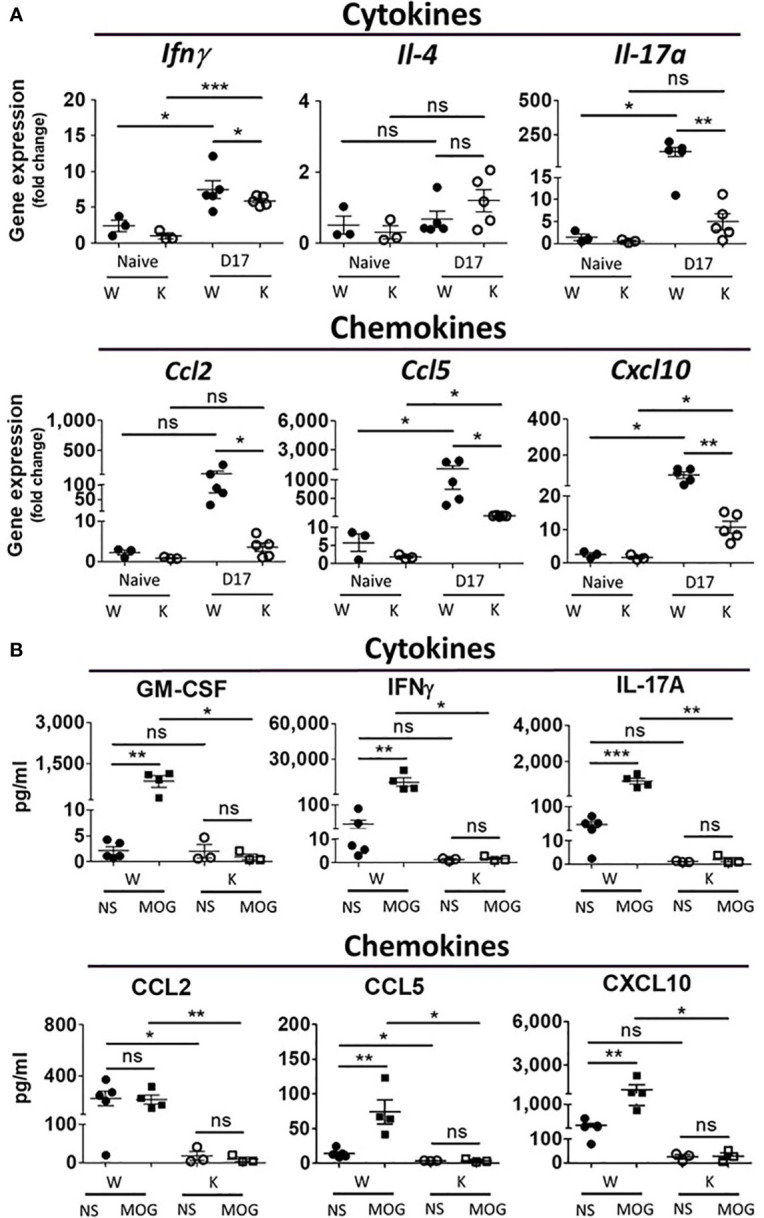
Nox2 deficiency diminishes EAE-elicited increase in mRNA expression in the spinal cord and secreted proteins of cytokine and chemokines from CNS mononuclear cells on disease peak (17 dpi). **(A)** mRNA levels (mean ± SEM) of spinal cord (L4-level section, 0.5 μm thick tissues) at 17 dpi after MOG inoculation were determined by RT-PCR. Cytokines: *Ifnγ, Il-4, Il-17a*; chemokines: *Ccl2, Ccl5, Cxcl10*. All data were normalized as fold of Naïve-W group. (n = 3 to 5 for each group) **(B)** The amount of cytokines (GM-CSF, IFNγ, IL-17A) and chemokines (Ccl2, Ccl5, Cxcl10) secreted from primary mononuclear cells isolated from the CNS collected at 17 dpi after MOG inoculation were quantified by ELISA assays. (n = 3 to 5 for each group). Cell culture condition was described in the results. NS, no drug treatment; MOG: 10 μg/ml. *p* value: *** < 0.001, ** < 0.01, * < 0.05, ns, not significant.

To determine the cytokines/chemokines gene expression profile in the spinal cord and the protein levels from isolated CNS monocytes, we measured levels of cytokine and chemokine-encoding mRNA in the spinal cord (L4-level section) as well as the levels of cytokines and chemokines secreted from cultured monocyte isolated from the CNS at 17 dpi. MOG treatment increased mRNA levels of cytokines *Ifnγ* (T_H_1) and *Il-17a* (T_H_17) as well as chemokines *Ccl2*, *Ccl5*, *Ccl6*, and *Cxcl10* in W mice. However, Nox2 deficiency weakened these MOG-elicited immune responses (**Figure 5A** and **Supplementary Figure 1A**). By contrast, the levels of *Il-4* and *Ccl20* mRNAs manifested minimum differences between the W and K MOG-injected mice (**Supplementary Figure 1A**). We also utilized ELISA to quantify the levels of cytokines (GM-CSF, IFNγ, TNFα, IL-1β, IL-6, IL-10, IL-12p70, and IL-17A) and chemokines (CCL2, CCL5, CXCL10) secreted from primary CNS-isolated mononuclear cells at 17 dpi (**Figure 5B** and **Supplementary Figure 1B**). To enhance the production of cytokines and chemokines from these cells, MOG (10 μg/ml) was added to their culture media for 3 days. MOG treatment caused a large increase in the supernatant levels of GM-CSF, IFNγ, TNFα, IL-6, IL-10, IL-17A, CCL2, CCL5, and CXCL10 produced by CNS monocytes isolated from W mice, but not those isolated from K mice. In contrast, no difference was observed in the levels of IL-1β or IL-12p70 secreted by CNS monocytes isolated from these mouse strains (**Figure 5B** and **Supplementary Figure 1B**).

To investigate the immune response in the spleen of our MOG-injected mice, we first used the ELISpot assay to quantify the levels of IFNγ, IL-4, and IL-17A secreted from total splenocytes at 7 dpi (before any clinical presentation) and 17 dpi (disease peak). The W and K groups showed no significance was found between Nox2-deficent and wildtype control groups (**Supplementary Figure 2A**). We then used an ELISA assay to quantify the amount of cytokines (GM-CSF, IFNγ, TNFα, IL-1β, IL-6, IL-10, IL-12p70, and IL-17A) and chemokines (CCL2, CCL5, and CXCL10) secreted from these splenocytes at 17 dpi. However, we were unable to detect any difference in levels of cytokines and chemokines secreted by splenocytes isolated from our mouse strains (**Supplementary Figure 2B**).

Collectively, these gene expression and protein level of cytokines and chemokines profiles in spleen are similar in both groups, while great reduction was found in Nox2-deficient mice in CNS. Therefore, not only the number of autoreactive T cells and myeloid cells showed great reduction in Nox2-deficient mice, but also similar trend was found in the cytokines and chemokines production in CNS on disease peak.

### Nox2 Deficiency Largely Reduces the Levels of Oxidative Stress in EAE Mice

**Figure 6:**
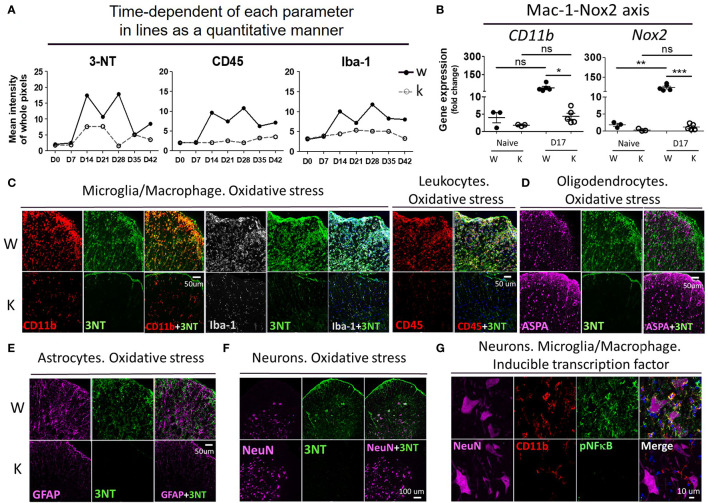
Nox2 deficiency largely reduces the production of oxidative stress and the signal of pNF-κB on disease peak (17 dpi) at spinal cord area. **(A)** Time-dependent of each parameter in lines as a quantitative manner. The mean fluorescence intensity of whole pixels was measured by Image J. **(B)** mRNA levels (mean ± SEM) of spinal cord (L4-level section, 0.5 μm thick tissues) at 17 dpi after MOG inoculation were determined by RT-PCR. Cell membrane protein: *CD11b* and *Nox2*, **(C)** The relationship between 3-NT (3-Nitrotyrosine, oxidative stress marker) signal and Iba-1 (microglia/macrophage), CD45 (leukocytes), CD11b (microglia/macrophage), **(D)** ASPA (aspartoacylase, active oligodendrocytes), **(E)** GFAP (astrocytes), **(F)** NeuN (total neurons). **(G)** The relationship between pNF-κB (inducible inflammatory transcription factor) and NeuN, CD11b. Genotype: W, *Nox2* ^+/+^; K, *Nox2* ^−/−^. Formalin-fixed frozen section: 10 μm. Spinal cord level: lumbar fourth. **(C–F)** marginal and ventral area of spinal cord (white matter); **(G)** ventral area of spinal cord (gray matter). *p* value: *** < 0.001, ** < 0.01, * < 0.05, ns, not significant.

To determine the effect of Nox2-deficincy on the levels of oxidative stress experienced in EAE mice in the CNS, we examined levels of 3-Nitrotyrosine (3-NT), the leukocyte marker CD45, and the microglia/macrophage marker Iba-1 in MOG-injected W and K mice over a period of 6 weeks (**Figure 6A** and **Supplementary Figure 3A**) in the spinal cord (L4 level). The W mice showed an abrupt increase of all these markers at 14 dpi, while the K mice showed little changes in the levels of these markers at this time. The 3-NT signal lasted for 2 weeks and subsided after 28 dpi, while the immunoreactivity of CD45 and Iba-1 remained elevated up to 42 dpi in W mice. It is important to note that MOG inoculation greatly increased the mRNA expression of two key superoxide free radical-producing molecules, *Nox2* and *CD11b* (a-chain of Mac-1 receptor), in W mice from the spinal cord tissue (**Figure 6B**).

To determine cell type responsible for increasing the oxidative stress levels in EAE mice, we performed doubling-immunostaining of 3-NT and markers for different cell types: Iba-1 and CD11b for microglia/macrophage, CD45 for leukocytes, aspartoacylase (ASPA) for active mature oligodendrocytes, GFAP for astrocytes, and NeuN for neurons (**Figures 6C–F**). In MOG-treated W mice, high immunoreactivity of 3-NT was observed in cells expressing CD45, CD11b, and Iba-1; to a lesser degree in NeuN-positive cells, but not in either ASPA- or GFAP-positive cells. In contrast, little detectible 3-NT immunoreactivity was found in all the cell types mentioned above in MOG-treated K mice. Mean fluorescent intensity of immunofluorescent slides: 3-NT (W: 10.16 ± 0.51, K: 5.24 ± 0.21, *p *< 0.001), CD45 (W: 9.80 ± 0.66, K: 1.81 ± 0.33, *p* < 0.001), CD11b (W: 12.58 ± 1.11, K: 3.74 ± 0.34, *p *< 0.01), Iba-1 (W: 10.29 ± 1.10, K: 1.76 ± 0.59, *p* < 0.01), NeuN (W: 8.78 ± 1.26, K: 10.14 ± 0.74, *p*: not significant), ASPA (W: 7.70 ± 0.52, K: 9.40 ± 0.25, *p* < 0.05), and GFAP (W: 10.44 ± 0.58, K: 5.392 ± 0.65, *p* < 0.01).

Regarding the costaining between Nox2 level and Iba-1, CD45, CD11b, CD16/32 (proinflammatory marker of microglia/macrophage/granulocytes) (28), and NeuN (a neuronal marker) at 17 dpi (**Supplementary Figures 3B, C**), Nox2 signal was mostly detected in CD45, CD11b, and Iba-1 positive cells, while it was almost undetectable in NeuN positive cells. Moreover, CD16/32, M1 activation marker, was abundant at CD45 population, which implies the presence of an active inflammatory response at the peak of disease. Mean fluorescent intensity of immunofluorescent slides: Nox2 (W: 12.15 ± 1.02, K: 1.54 ± 0.63, *p* < 0.001), CD16/32 (W: 8.31 ± 0.55, K: 1.68 ± 0.37, *p* < 0.001).

Since ROS activates NF-κB signaling, which modulates gene expression in diverse cellular processes such as innate immune response (29, 30), we next determined if NF-κB plays were required for the expression of inflammatory transcription factors in our EAE model. To do this, we co-stained the active form of NF-κB (pNF-κB) with NeuN or CD11b (**Figure 6G**). The W mice exhibited an abundant pNF-κB signal, which partially colocalized with CD11b, but not with NeuN. Yet, the K mice showed almost no pNF-κB signal at 17 dpi. Mean fluorescent intensity of immunofluorescent slides: pNF-κB (W: 11.87 ± 1.06, K: 3.62 ± 0.22, *p* < 0.01).

### Nox2 Deficiency Diminishes the EAE-Elicited Increase in Microglia Activation as Well as Cytokine/Chemokine Secretion in the CNS

**Figure 7:**
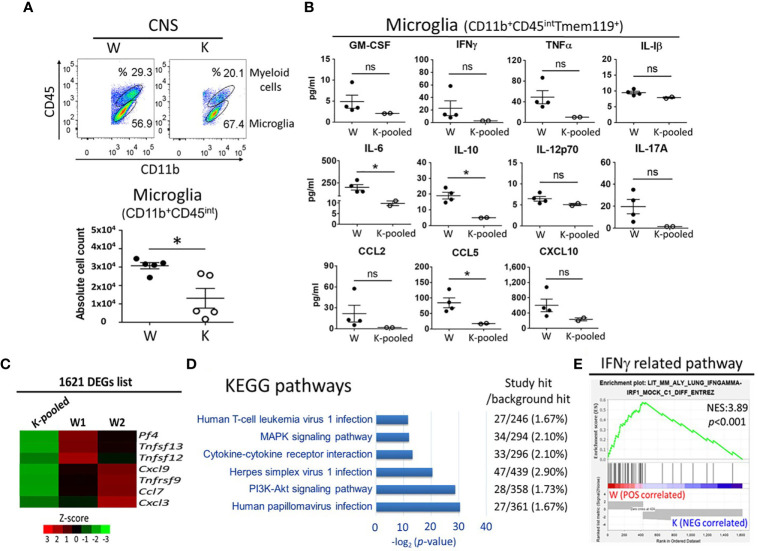
Cytokine/chemokine and transcriptome profiles of Nox2-deficient microglia. CD11b^+^CD45^int^Tmem119^+^ microglia were isolated from the CNS tissue of Nox2-competent and deficient mice on disease peak (17 dpi). **(A)** microglia (CD11b^+^CD45^int^) count analyzed by flow cytometry (n = 5 each group). **(B)** Concentrations of cytokines and chemokines released from primary cultured microglia. [n = 4 (W), 2 (K-pooled)]. MOG: 10 μg/ml. *p* value: * < 0.05; ns, not significant. **(C)** Relative mRNA levels of cytokine/chemokine genes in isolated microglia as determined by RNAseq. Color scales depict relative fold changes. **(D)** Significantly enriched KEGG pathways in association with immune response, inflammation, infection, and known EAE related pathways. **(E)** Correlations of the IFNγ-related pathway with Nox2-competent and deficient microglia transcriptome GSEA. *p* value: * < 0.05; ns, not significant.

To investigate the specific contribution of microglia at this model, we utilized flowcytometry analysis showed that a 58.8% decrease in the number of microglia (CD11b^+^CD45^int^) (20, 31) taken at 17 dpi in K mice, as compared to controls (**Figure 7A**). Sorted microglia (CD11b^+^CD45^int^Tmem119^+^, purity >95%) (32) from each group were then seeded in a density of 3 × 10^4^/500μl/well. Following 42 h incubation, supernatants from primary microglia culture were collected for ELISA analysis (**Supplementary Figure 4**). The data show that levels of IL-6, IL-10, and CCL5 were lower in the MOG-injected K mice relative the MOG-injected W mice (**Figure 7B**). However, levels of GM-CSF, IFNγ, TNFα, IL-17A, CCL2, and CXCL10 showed a trend of decrease in the K group compared to the W group. Nevertheless, these differences were not statistically significant. likely due to the small sample sizes. No difference was found for the levels of IL-1β and IL-12p70 between MOG-injected W and K mice (**Figure 7B**). Thus, Nox2-deficient microglia failed to secret enough cytokines and chemokines to induce the invasion of peripheral pathogenic immune cells into the CNS.

### Nox2 Deficiency Affects Multiple Cytokine/Chemokine- and Inflammation-Related Pathways in Microglia

Next, we performed RNAseq to determine the global impact of Nox2 deficiency on microglia functions and to identify Nox2 associated pathways. Genome-wide transcriptome profiling identified 1621 differentially expressed genes (DEG) with ≥ 3-fold changes between W and K microglia cells treated with exogenous MOG (**Supplementary Figure 4** and **Supplementary Table 2**). In addition to the aforementioned changes in secreted cytokines and chemokines (**Figure 7B**), expression of chemotactic gene *Pf4* (33) and proinflammatory cytokines and chemokines *Tnfrsf9*, *Tnfsf12*, *Tnfsf13*, *Ccl7*, *Cxcl3*, and *Cxcl9* (34–39) also showed a reduction in response to Nox2 deficiency (**Figure 7C**). These results indicate that Nox2 plays a broad role in regulating the activation of microglia (1, 40). Gene ontology analysis performed on the KEGG pathways (**Supplementary Table 3**) revealed DEG enrichment in six pathways that are associated with virus infection (human papillomavirus infection, herpes simplex virus 1 infection, and human T-cell leukemia virus 1 infection), which are known to be associated with EAE-related signaling pathways, such as the MAPK and PI3K-Akt signaling pathways as well as cytokine-cytokine receptor interaction (**Figure 7D**). Ingenuity Pathway Analysis further identified an enrichment of the STAT3 pathway (41), glutathione-mediated detoxification (42), leukotriene biosynthesis (43), IL-8 signaling (44), HMGB1 signaling (45), NRF2-mediated oxidative stress response (46), systemic lupus erythematosus in B cell signaling pathway (47), and T cell exhaustion signaling pathway (48) in the Nox2 gene signature and are in association with MS or EAE (**Table 1** and **Supplementary Table 4**). Importantly, Gene Set Enrichment Analysis (GSEA) found that 35 out of 42 significantly enriched gene sets from Nox2-competent microglia are inflammation or infection-related. However, none of the 13 gene sets identified in Nox2-deficient microglia belong to these categories (**Supplementary Table 5**), as exemplified by the IFNγ-related pathway (**Figure 7E**). Collectively, our findings suggest that Nox2 is required for the activation of and cytokine/chemokine secretion by microglia, which has important implications on the neuroinflammation process in EAE.

**Table 1:** Nox2 dependent pathways in microglia with an association with multiple sclerosis or experimental autoimmune encephalomyelitis (EAE).

Pathway	*p* value (-log10)	References
STAT3 Pathway	4.44	Hsueh Chung Lu et al. STAT3 signaling in myeloid cells promotes pathogenic myelin-specific T cell differentiation and autoimmune demyelination. *Proc Natl Acad Sci* U S A. 2020 Mar 10; 117(10): 5430-5441 (41).
Glutathione-Mediated Detoxification	2.98	Andreia N Carvalho et al. Glutathione in multiple sclerosis: more than just an antioxidant? *Mult Scler*. 2014 Oct; 20(11):1425-31 (42).
Leukotriene Biosynthesis	2.94	Liefeng Wang et al. Antiasthmatic Drugs Targeting the Cysteinyl Leukotriene Receptor 1 Alleviate Central Nervous System Inflammatory Cell Infiltration and Pathogenesis of Experimental Autoimmune Encephalomyelitis. *J Immunol* 2011; 187:2336-2345 (43).
IL-8 Signaling	2.65	Takaaki Ishizu et al. Intrathecal activation of the IL-17/IL-8 axis in opticospinal multiple sclerosis. *Brain*. 2005 May; 128(Pt 5):988-1002 (44).
HMGB1 Signaling	2.41	Andrew P Robinson et al. High-mobility group box 1 protein (HMGB1) neutralization ameliorates experimental autoimmune encephalomyelitis. *J Autoimmun*. 2013 Jun; 43:32-43 (45).
NRF2-Mediated Oxidative Stress Response	2.34	Itzy E. Morales-Pantoja et al. Nrf2-dysregulation correlates with reduced synthesis and low glutathione levels in experimental autoimmune encephalomyelitis. *J Neurochem.* 2016 Nov; 139(4): 640–650 (46).
Systemic Lupus Erythematosus in B Cell Signaling Pathway	1.47	Qianxia Zhang and Dario A A Vignali. Co-stimulatory and Co-inhibitory Pathways in Autoimmunity. *Immunity*. 2016 May 7;44(5):1034-51 (47).
T Cell Exhaustion Signaling Pathway	1.3	Norio Chihara. Dysregulated T cells in multiple sclerosis. *Clin. Exp. Neuroimmunol.* 9 (Suppl. 1), (2018) 20–29 (48).

Pathways were identified by the Ingenuity Pathway Analysis based on the 1621 DEGs.

## Discussion

**Figure 8:**
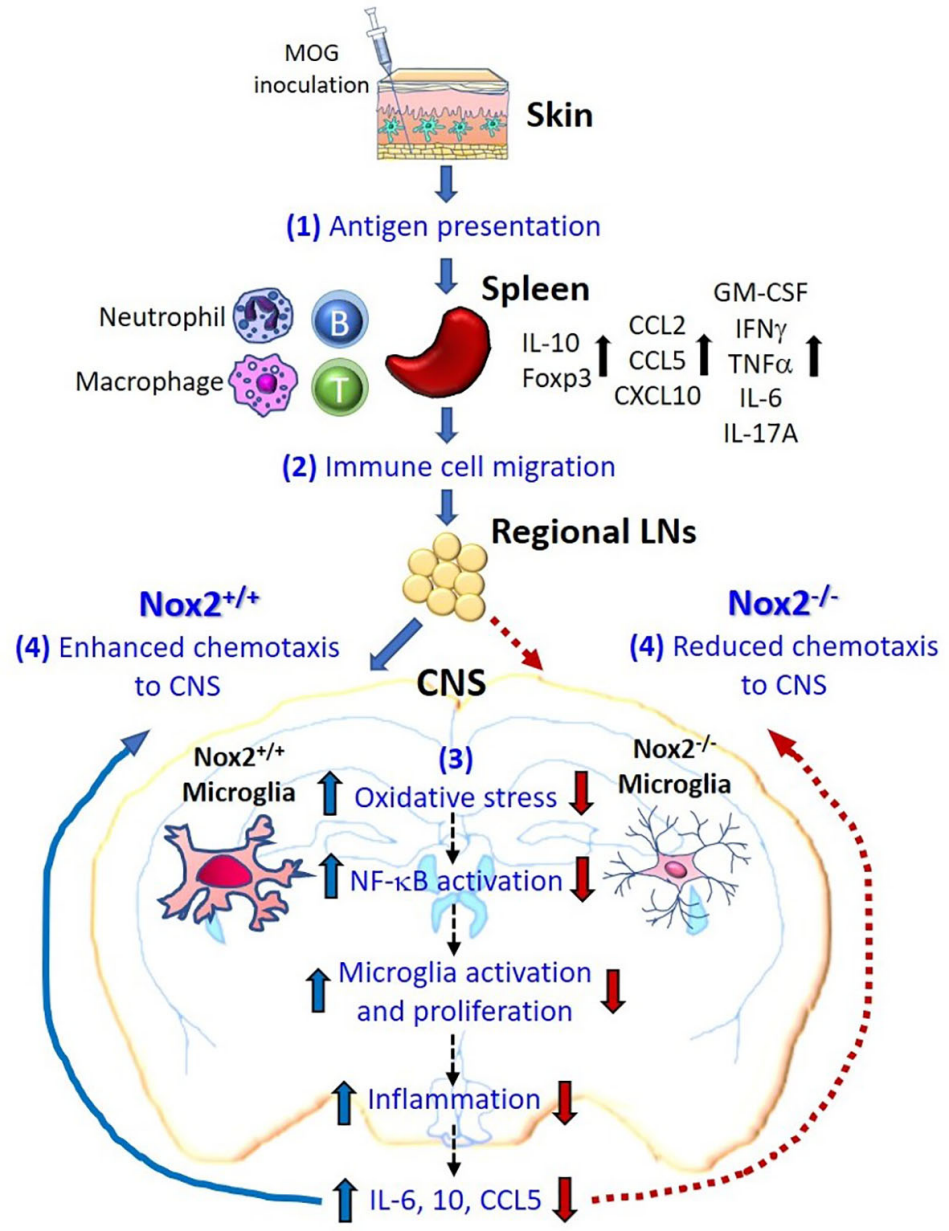
Schematic drawing depicting how microglial Nox2 promotes the pathogenesis of EAE. Our study suggests that Nox2-mediated oxidative stress and chemotaxis in microglia play an important pathophysiological role in EAE-induced neuronal damage as shown in the following stages. (1) Initially, subcutaneous MOG immunization recruits mainly local dendritic cells and, in partly, local macrophages to recognize and uptake the MOG antigen. Thereafter, these cells migrate to nearby inguinal lymph nodes and activate autoreactive T cells through antigen presentation. These primed and activated immune cells further migrate to the spleen where subsequently augment the proliferation of these immune cells as well as the production of multiple cytokines or chemokines. (2) Various immune cells producing different cytokines and chemokines further migrate to the regional lymph nodes near CNS territory. (3) The Nox2-deficient microglia fail to produce ROS in response to peripheral immune reaction and in turn reduce the downstream chain reactions, like NF-κB activation, microglia activation and proliferation, inflammation, and production of cytokines and chemokines (IL-6, IL-10, CCL5). (4) Summation of all the above responses, Nox2-deficient microglia effectively reduce the infiltration of pathogenic leukocytes to CNS through reduced chemotaxis ability, which subsequently blocked the immune cells from invading into the CNS in the final step. In contrast, Nox2-competent microglia exhibit an enhanced chemotaxis function. In conclusion, our results demonstrated that the oxidative stress, inflammatory reaction, and chemotaxis from Nox2-deficient mice are largely reduced in the CNS which greatly halted the neurodegeneration process.

This study reveals a critical role for Nox2 in the induction of MOG-elicited EAE in mice. Our results strongly suggest that the superoxide-producing enzyme Nox2 is essential for the activation of microglia, which is critical for their ability to cause persistent neuroinflammation. Furthermore, gene ontology and pathway enrichment analyses indicate a regulatory role of microglial Nox2 in multiple pathways associated with MS/EAE, particularly the chemotactic factor, *Pf4* (33). This result indicates that one of the important functions performed by microglial Nox2 is to increase the chemotaxis of peripheral pathogenic immune cells into the CNS. Consequently, all the recruiting immune cells, including autoreactive T cells and CNS-infiltrating myeloid cells along with microglia, synergistically augment the inflammatory process (**Figure 8**). Taken together, our results provide new mechanistic insights into the contribution of Nox2 and therefore oxidative stress to the pathogenesis of EAE and suggest that Nox2 inhibition may be a promising therapeutic target for MS.

### Nox2, a Key Superoxide-Producing Enzyme, Play a Critical Role in MOG-Induced EAE Mice

Although many enzymes can produce superoxide/ROS [e.g. xanthine oxidase, lipoxygenase, cyclooxygenase, cytochrome P450, nitric oxide synthetase, and NADPH oxidase (49)] and are responsible for different redox regulations in various immune responses (50), our results suggest that Nox2 is the key enzyme for the excessive production of ROS after MOG autoantigen challenge in EAE model. Even though neuroinflammation is most likely a primary reaction of the host aimed at removing invading pathogens and initiating healing processes (51), excessive and prolonged neuroinflammation may be detrimental to neuronal and oligodendrocyte cells and thus promotes the progression of EAE.

Several previous reports have investigated the role of Nox2 in EAE. It was first shown that Nox2-deficiency affected the ability of bone marrow-derived macrophages to process antigens and to induce subsequent T_H_ cell-driven disease process in MOG-elicited EAE model (52). Furthermore, Nox2-regulated MOG-antigen processing in conventional dendritic cells (cDC) licenses encephalitogenic T_H_ cells to initiate autoimmune neuroinflammation (53). It is important to point out that we used conventional Nox2 KO mice in this experiment, thus, Nox2 is also deficient in DC and macrophage in this KO mice. Therefore, we can’t rule out potential contributions from DC and macrophage and other immune cells in this study. Instead, our study provided additional complimentary function by illustrating potential interactions of microglia with peripheral immune cells. Keller et al. (53) not only used conditional KO mice (*cybb^fl/fl^-Itgax-Cre* and *cybb^fl/fl^-Zbtb46-Cre*) in their study, they further utilized adoptive transfer model and focused on cDC population as their investigational interest. However, we found that the peripheral T cell activation and proliferation are similar in the spleen and cervical lymph nodes between Nox2 KO and wildtype mice in our active EAE model, while Keller et al. showed similar data that no difference of T cell proliferation and activation between *cybb^fl/fl^-Itgax-Cre* compared with *cybb^fl/fl^* littermates. In addition, Nox2-deficincy may lead to clinical improvement potentially due to elevated anti-inflammatory cytokines, IL-4 and IL-10 (54, 55). However, we found that production of cytokines of IL-4 and IL-10 in the CNS were lower in the Nox2 KO mice in our study. The results were inconsistent with the reports of Hernández-Espinosa’s and Ravelli’s articles (54, 55). A possible explanation for this inconsistency is due to different CNS regions were used. Ravelli et al. (55) used striatum and motor cortex to perform gene expression (*Il-4*, *Il-10*) and protein level (IL-10) while our study used samples from L4-spinal cord (immune cells infiltration hot spot, near MOG inoculation site). It is likely that the degree of immune cell infiltration is higher in the spinal cord regions than that in the brain. Thus, the source of IL-4, IL-10 in the spinal cord could derived from a more variety of immune cells. Since many immune cells can secret IL-4 and IL-10, the results are the summation of mixed cell populations, which may cause the difference. A translational study reported that dextromethorphan, a widely used antitussive medicine, effectively ameliorated the severity of EAE through its anti-inflammatory effect by repressing Nox2-dependent superoxide production in activated microglia (56).

Current MS therapeutics mainly target the adaptive immune system; however, they are not particularly effective in halting the disease progression. Recent studies have begun to focus on the innate immunity system in EAE model. Our study provides significant insights into the role of microglial Nox2 in initiating and introduction of MOG-elicited EAE model and how over-activation of Nox2 producing the immune dysfunction leading to chronic inflammation and neurodegeneration.

### Microglial Nox2 Gene signature and Involved Pathways in EAE

Accumulating evidence indicates that deregulated microglial Nox2 activity is a key mechanism in a variety of neurodegenerative diseases, such as Parkinson’s (57) and Alzheimer diseases (58). In EAE model, we believe Nox2 promotes leukocyte infiltration and demyelination by producing superoxide and its related ROS. Sakai et al. (59) reported that different species of ROS are involved in neutrophil polarization, chemotaxis, adhesion, and phagocytosis. Our previous report (60) also indicated that activation of Nox2 and translocation of the Nox2 p47*^phox^* subunit to the plasma membrane are vital steps in initiating α-syn–induced microglial directional migration. Thus, it is highly likely that activation of microglia Nox2 activity in an EAE model is involved in the infiltration of leucocytes. It is also well documented that over-production of superoxide/ROS by activation of NOX2 can enhance oxidative stress and leading to demyelination (61). Hence, over-activation of this superoxide-producing enzyme underlies the initiation of acute inflammation and maintenance of low-grade chronic inflammation in the CNS (62). Prior study extends previous findings to include MS (63). Although compared with either Parkinson’s or Alzheimer’s Disease, many more types of immune cells are involved in the pathogenesis of MS, our study suggests that microglial Nox2 is critically involved in the initiation and introduction of EAE pathologies.

The contribution of over-activated microglial Nox2 to chronic neuroinflammation that leads to neurodegeneration has been extensively studied. Briefly, over-production of superoxide and its ROS metabolites enhances the expression of proinflammatory immune factors such as TNFα, IL-6, prostaglandins, etc. that initiate inflammation (64). Moreover, formation of peroxynitrite through the reaction of superoxide with nitric oxide is toxic to oligodendrocytes and neurons (65). Furthermore, reports from our laboratory and others indicate that Nox2-generated superoxide/H_2_O_2_ is a potent mediator of immune cell chemotaxis (66). These results are consistent with our current finding from the analysis of gene profile using primary microglia cultures isolated from disease peak stage of EAE mouse. Gene profile analysis showed that the most different DEGs signature between Nox2^−/−^ and wildetype control is the chemotactic gene *Pf4*. This gene product has a strong chemotactic effect on monocytes as well as neutrophils and forms a heterodimer with CCL5 that promotes the migration of monocytes (33). The proinflammatory cytokines and chemokines genes *Tnfrsf9, Tnfsf12, Tnfsf13, Ccl7, Cxcl3*, and *Cxcl9* also emerged in the significantly different DEGs list and showed a reduction in response to Nox2 deficiency. In addition, our IPA analysis infers that the STAT3 pathway (41) is regulated by *Nox2*. Deregulation of STAT3, a transcription factor critical for T_H_17 cell differentiation (67), has been implicated in MS/EAE. Previous research pointed out that the activation of STAT3 in myeloid cells is essential for leukocyte infiltration, neuroinflammation, and demyelination in EAE mice (41), while phosphorylated STAT3 was also observed in microglia, macrophages, and astrocytes in the white matter adjacent to active MS lesions (68). Collectively, these findings support the role of Nox2 in microglia as a key mediator of chemotaxis during the early stage of EAE.

### Nox2 Deficiency Specifically Affects the CNS Immune System

It is interesting to point out that Nox2-deficiency may show variable outcomes in different disease models. For example, using rheumatoid arthritis model (69, 70) reported that Nox2-deficiency aggravates disease process, while our EAE model indicates that Nox2-deficiency protects mice from disease onset and progression. Further, Nox2 deficiency affects the CNS, but not the peripheral immune systems. The arthritis model from Lee et al. (69) showed Nox2-deficient mice developed spontaneous arthritis while wildtype mice remain healthy. The immune response in the spleen and regional lymph nodes showed dramatic increase in Th17 cells development as well as Th1 and CD11b^+^Gr-1^+^ myeloid cells and decrease in Treg cells in Nox2 KO mice. Contrarily, our study found that the numbers of infiltrated T cells (Th1, Th17, Treg) and myeloid cells were simultaneously increased in the peripheral lymphoid organs (spleen and cervical lymph nodes) between MOG-injected Nox2 KO and wildtype mice at disease peak, while their numbers were greatly decreased in the CNS of Nox2 KO mice. Additionally, the gene expression and protein levels of cytokines and chemokines from spleen showed no difference at disease peak between Nox2 KO and wildtype mice, while dramatic differences were observed in the CNS between these groups.

Some of our findings are consistent with several previous reports. Allan et al. (52) reported that Nox2-deficiency won’t affect the ability of bone marrow-derived dendritic cells (BMDCs), but bone marrow-derived macrophage (BMDMs) to process and present MOG antigen in an active EAE model. Keller et al. (53) showed that in a DC-specific Nox2 KO mutant mouse, the T cell proliferation, activation as well as cytokines production are similar between KO and wild type mice in an adoptive transfer EAE model. Cachat et al. (71) reported that Nox2-deficent T cells co-cultured with wildtype BMDCs do not impact T cell activation or proliferation *in vitro* and *in vivo*. Similarly, Nox2-deficient BMDCs cocultured with wildtype T cells showed mild effect on T cell activation, but not on their proliferation index. Taken together, Nox2 deficiency does not seriously impact antigen presentation, T cell activation, or the peripheral immune response induced by MOG injection in our EAE model. These results indirectly support our findings that microglial Nox2 is a critical driving force for peripheral pathogenic immune cells to gain access to the CNS and is the critical element in the successful induction of MS/EAE.

## Conclusion

In summary, we have provided strong evidence indicating that deletion of Nox2 can sufficiently impair ROS production and reduce oxidative damage. Furthermore, our data suggest that microglial Nox2 plays an important role in chemotaxis in recruiting activated peripheral pathogenic immune cells to the CNS territory. Finally, this study provides insights into a possibility that microglial Nox2 is a prime target for MS therapy.

## Data Availability Statement

The datasets presented in this study can be found in online repositories. The names of the repository/repositories and accession number(s) can be found below: https://www.ncbi.nlm.nih.gov/, BioProject number: PRJNA668563.

## Ethics Statement

The animal study was reviewed and approved by the committee of Institutional Animal Care and Use Committee of the National Defense Medical Center (IACUC: 19-058).

## Author Contributions

C-FH, S-PW, S-HC, S-JC, and J-SH: conceptualization. C-FH, S-PW, C-SH, C-CS, and S-JC: methodology. C-FH, J-WC, and S-PW: software. G-JL and C-CS: validation. C-FH, S-PW, and C-SH: formal analysis. C-FH and S-PW: investigation. J-SH and S-JC: resources. G-JL, C-CS, J-WC, and S-HC: data curation. C-FH: writing—original draft. S-PW, G-JL, J-SH, and S-JC: writing—review and editing, J-SH and S-JC: supervision. C-FH and S-JC: project administration. C-FH and S-JC: funding acquisition. All authors contributed to the article and approved the submitted version.

## Funding

This research was funded by Tri-Service General Hospital, grant number: TSGH-C107-016, TSGH-C108-021, TSGH-D-109037 (C-FH); TSGH-C108-007-008-S03, TSGH-C01-109014 (S-JC) and by Ministry of Science and Technology, grant number: MOST106-2314-B-016-041-MY3 (S-JC). This work is supported in part by an Intramural Research Program of the National Institute of Environmental Health Sciences, National Institutes of Health Z99-ES999999 (S-PW).

## Conflict of Interest

The authors declare that the research was conducted in the absence of any commercial or financial relationships that could be construed as a potential conflict of interest.
