# Supplementary material for: Microglial Nox2 Plays a Key Role in the Pathogenesis of Experimental Autoimmune Encephalomyelitis
Source: Front Immunol. 2021 Apr 2;12:638381. doi: 10.3389/fimmu.2021.638381 (PMC8050344; doi:10.3389/fimmu.2021.638381)
Supplement: Supplementary file 1 [file DataSheet_1.docx]

Supplement data

**
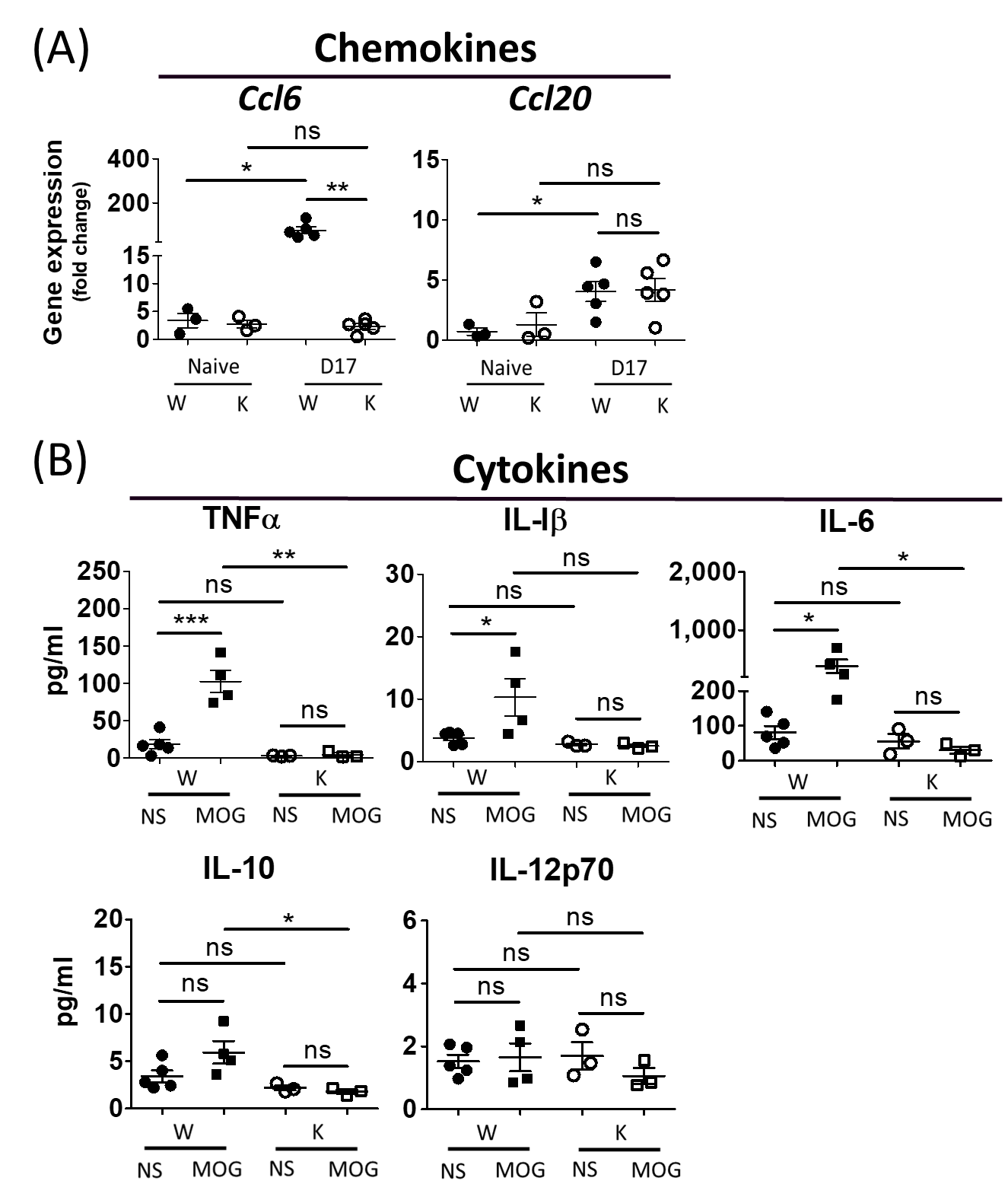
**

**Figure S1. The mRNA expression from spinal cord tissue and the secretion of cytokines from cultured CNS monocyte cells respectively on disease peak (day 17).** (A) the mRNA level (mean±SEM) of spinal cord (L4-level section, 0.5μm thick tissuse) at day 17after MOG inoculation were determined by RT-PCR. Chemokines: *Ccl6, Ccl20*. All data were normalized as fold of Naïve-W group (n = 3 to 5 for each group). (B) The amount of cytokines (TNFα, IL-1β, IL-6, IL-10, IL-12p70) secreted from primary mononuclear cells from CNS tissue collected at day 17 after MOG inoculation were quantified by ELISA assays. (n = 3 to 5 for each group). Cell culture conditon was described in the results. NS: no drug treatment, MOG: 10 μg/ml. *p* value: ***:<0.001, **:<0.01, *:<0.05, ns: not significant.

**
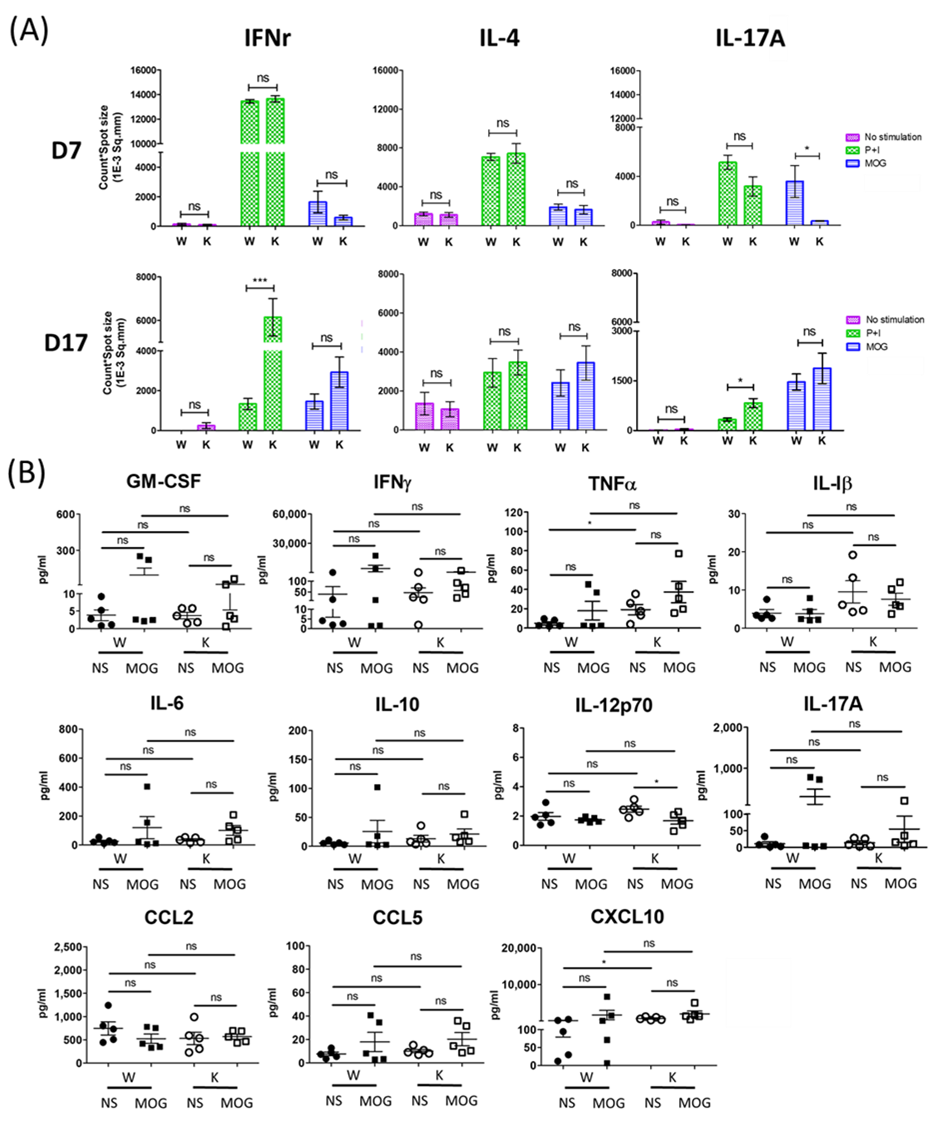
**

**Figure S2. Nox2-deficient and Nox2-competent mice show no significant difference in EAE-elicited cytokines and chemokines secretion from splenocytes on preclinical (day 7) and disease peak (day 17) stage.** (A) The enzyme-linked immunospot (ELISpot) assay for quantification of IFNγ, IL-4 and IL-17A secreted from cutured splenocyte cells on day 7 (before any clinical presentation) and day 17 (disease peak) respectively (n= D7: 6 (W), 6 (K); D17: 12 (W), 12 (K)). We allocated each genotype into three groups with different in vitro stimulation, including MOG (10 μg/ml) (antigen-dependent stimulation), PMA (2ng/ml)+ Ionomycin (0.1 μg/ml) (antigen-independent stimulation), and no stimulation. The Y-axis represents the number of cytokine secreting cell counts multiplied by spot size to objectively provide the ability of cytokines secretion (mean±SEM). (B) The amount of cytokines (GM-CSF, IFNγ, TNFα, IL-1β, IL-6, IL-10, IL-12p70, IL-17A) and chemokines (CCL2, CCL5, CXCL10) secreted from splenocytes collected at day 17 after MOG inoculation were quantified by ELISA assays. (n= 5 for each group). Cell culture conditon was described in the results. NS: no drug treatment, MOG: 10 μg/ml. *p* value: ***:<0.001, **:<0.01, *:<0.05, ns: not significant.


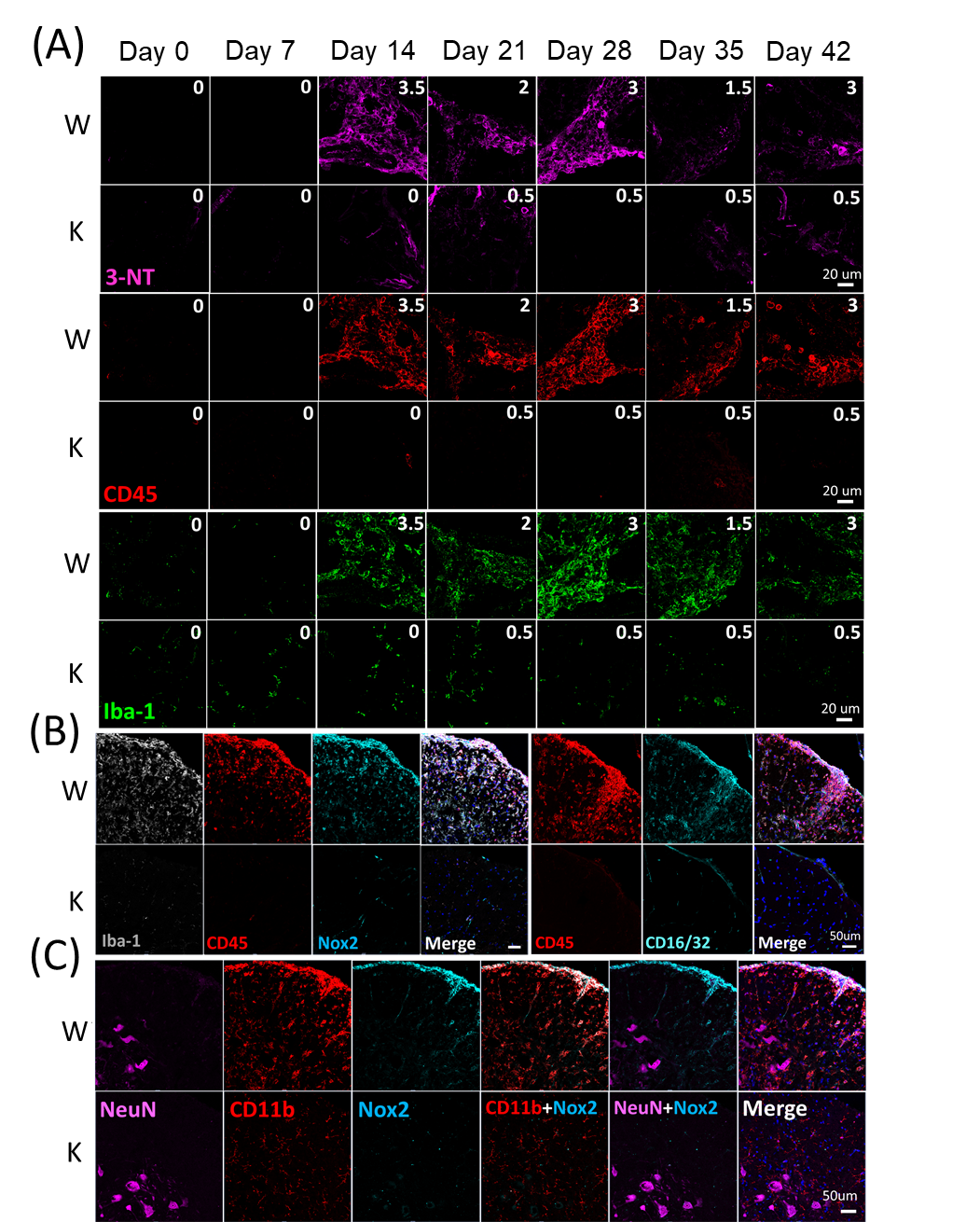


**Figure S3. Nox2-deficiency greatly reduced EAE-elicited increase in the production of oxidative stress, leukocytes infiltration, and microglia in a time dependent manner.** (A) The weekly signal changes of oxidative stress (3-NT), leukocytes (CD45), and microglia/macrophage (Iba-1) during six weeks of disease course. Right upper corner showed clinical EAE score. (B-C) The relationship between Nox2 signal and Iba-1, CD11b (microglia/macrophage), CD45 (leukocytes), CD16/32 (proinflammatory subtype of microglia/macrophage/ granulocytes, M1 marker), and NeuN (total neurons) on disease peak (day 17). Formalin-fixed frozen section: 10μm. L4 spinal cord cross section at marginal and ventral area (white matter).

**
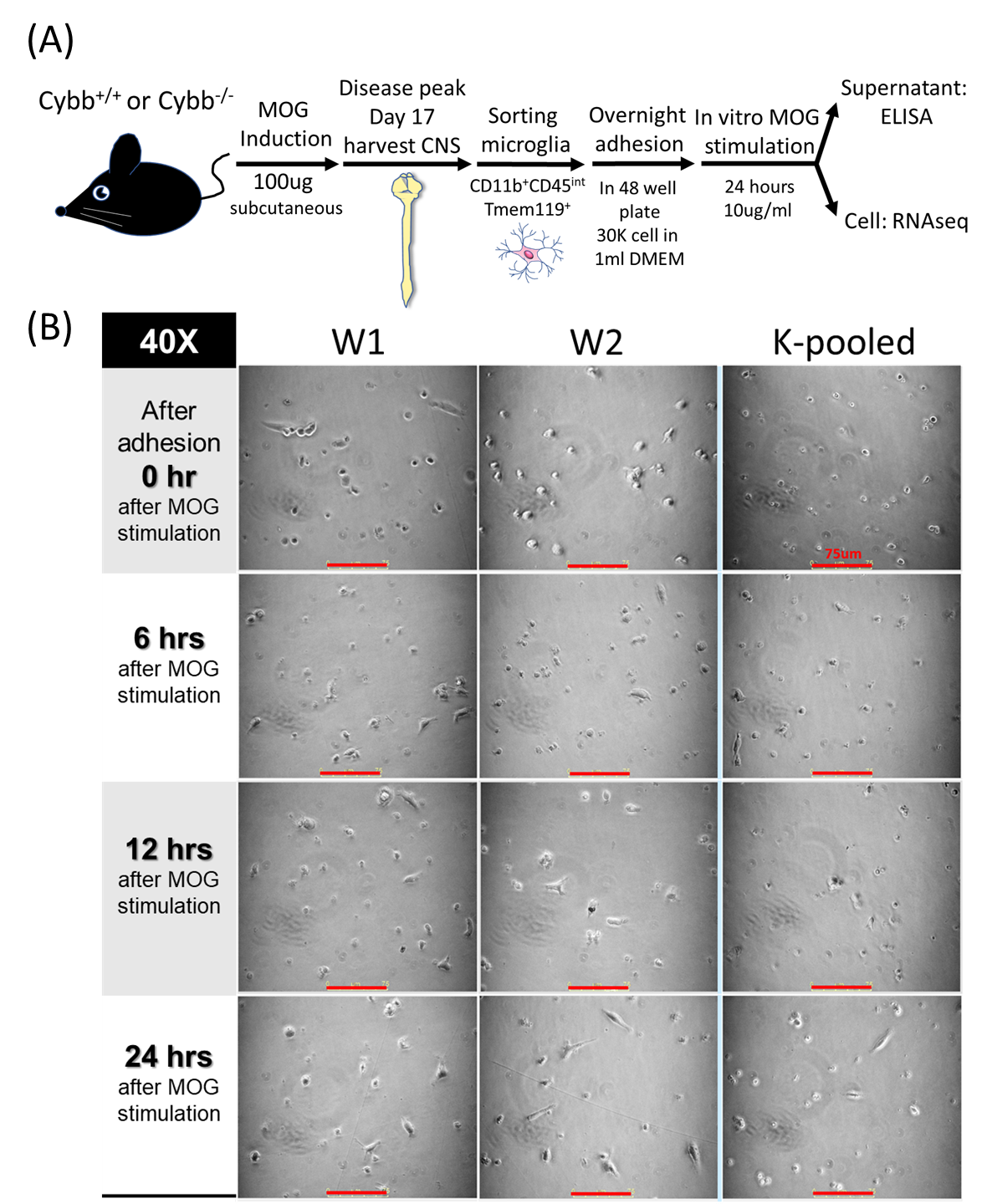
**

**Figure S4. The workflow of primary microglia culture (CD11b^+^CD45^int^Tmem119^+^) from disease peak stage (day 17) (A) and serial imaging before ELISA and RNAseq analyses (B).** We seeded these microglia in 3x10^4^/500ul/well and cultured them for 42 hours (18 hours of adhesion+24 hours of in vitro stimulation). MOG: 10 μg/ml. W1 and W2 are single mouse per sample while K-pooled mixed three mice in one sample. Scale bar: 75μm.
