# Supplementary material for: Microglial Nox2 Plays a Key Role in the Pathogenesis of Experimental Autoimmune Encephalomyelitis
Source: Front Immunol. 2021 Apr 2;12:638381. doi: 10.3389/fimmu.2021.638381 (PMC8050344; doi:10.3389/fimmu.2021.638381)
Supplement: Supplementary file 2 [file Table_1.docx]

| **Table S1. Description of primers used throughout the study to analyze the genes of interest** | | | |
| --- | --- | --- | --- |
| **Gene** | | **Primers sequence (5’ to 3’)** | |
|  |  | **Forward** | **Reverse** |
| Surface markers | *Nox2* | GATTCAAGATGGAGGTGGGAC | GGTCAGTGTGAATGGGTGCC |
|  | *CD11b* | ATGCGACAGTGAAAACCAAGGC | CTAAGGAGAGACCCCCAACAGC |
| Cytokines | *IFNγ* | AAGCGGCTGACTGAACTC | CTGTTACTACCTGACACATTCG |
|  | *IL-4* | GCTAGTTGTCATCCTGCTCTTC | TGGTGTTCTTCGTTGCTGTG |
|  | *IL-17A* | ACGTCACCCTGGACTCTC | TCCCTCCGCATTGACACA |
| Chemokines | *CCL2* | TTCACAGTTGCCGGCTGG | TGAATGAGTAGCAGCAGGTGAGTG |
|  | *CCL5* | CAGCAGCAAGTGCTCCAATCTT | TTCTTGAACCCACTTCTTCTCTGG |
|  | *CCL6* | TTGTGGCTGTCCTTGGGTC | AGTGTCTTGAAAGCCTTGATGAATT |
|  | *CCL20* | CTGCTGGCTCACCTCTGCA | CATCGGCCATCTGTCTTGTG |
|  | *CXCL10* | GAAATCATCCCTGCGAGCCT | TTGATGGTCTTAGATTCCGGATTC |
| Internal control | *GAPDH* | TTCAACGGCACAGTCAAGGC | GACTCCACGACATACTCAGCACC |
